# Mitochondrial-Derived Vesicles as Candidate Biomarkers in Parkinson’s Disease: Rationale, Design and Methods of the EXosomes in PArkiNson Disease (EXPAND) Study

**DOI:** 10.3390/ijms20102373

**Published:** 2019-05-14

**Authors:** Anna Picca, Flora Guerra, Riccardo Calvani, Cecilia Bucci, Maria Rita Lo Monaco, Anna Rita Bentivoglio, Francesco Landi, Roberto Bernabei, Emanuele Marzetti

**Affiliations:** 1 Università Cattolica del Sacro Cuore, Institute of Internal Medicine and Geriatrics, 00168 Rome, Italy; anna.picca1@gmail.com (A.P.); francesco.landi@unicatt.it (F.L.); 2 Fondazione Policlinico Universitario “Agostino Gemelli” IRCCS, 00168 Rome, Italy; mariarita.lomonaco@policlinicogemelli.it (M.R.L.M.); annarita.bentivoglio@unicatt.it (A.R.B.); emanuele.marzetti@policlinicogemelli.it (E.M.); 3 Department of Biological and Environmental Sciences and Technologies, University of Salento, 73100 Lecce, Italy; guerraflora@gmail.com (F.G.); cecilia.bucci@unisalento.it (C.B.); 4 Università Cattolica del Sacro Cuore, Institute of Neurology, 00168 Rome, Italy

**Keywords:** exosomes, mitophagy, mitochondrial quality control, mitochondrial-lysosomal axis, mtDNA, extracellular vesicles

## Abstract

The progressive loss of dopaminergic neurons in the nigro-striatal system is a major trait of Parkinson’s disease (PD), manifesting clinically as motor and non-motor symptoms. Mitochondrial dysfunction and oxidative stress are alleged pathogenic mechanisms underlying aggregation of misfolded α-synuclein that in turn triggers dopaminergic neurotoxicity. Peripheral processes, including inflammation, may precede and contribute to neurodegeneration. Whether mitochondrial dyshomeostasis in the central nervous system and systemic inflammation are linked to one another in PD is presently unclear. Extracellular vesicles (EVs) are delivery systems through which cells can communicate or unload noxious materials. EV trafficking also participates in mitochondrial quality control (MQC) by generating mitochondrial-derived vesicles to dispose damaged organelles. Disruption of MQC coupled with abnormal EV secretion may play a role in the pathogenesis of PD. Furthermore, due to its bacterial ancestry, circulating mitochondrial DNA can elicit an inflammatory response. Therefore, purification and characterisation of molecules packaged in, and secreted through, small EVs (sEVs)/exosomes in body fluids may provide meaningful insights into the association between mitochondrial dysfunction and systemic inflammation in PD. The EXosomes in PArkiNson Disease (EXPAND) study was designed to characterise the cargo of sEVs/exosomes isolated from the serum of PD patients and to identify candidate biomarkers for PD.

## 1. Introduction

Parkinson’s disease (PD) is the second most common age-related neurodegenerative disease, affecting 2–3% of the population aged 65+. PD is characterised by the progressive loss of midbrain dopaminergic neurons of the substantia nigra pars compacta, leading to motor (i.e., bradykinesia, postural inability, rigidity and tremor) and non-motor signs and symptoms (e.g., constipation, depression, sleep, cognitive dysfunction) [1]. Aggregation of misfolded α-synuclein triggering dopaminergic neurotoxicity is a well-established pathologic trait of PD. However, emerging evidence suggests that co-occurrence of peripheral changes (e.g., inflammation) might precede and contribute to neurodegeneration [2,3,4]. Though, the impact of these processes on disease onset and progression remains elusive.

Mitochondrial dysfunction has been associated with several neurodegenerative diseases, including PD, through increased oxidative stress favouring aberrant protein folding and accrual of protein aggregates (i.e., amyloid β, huntingtin, tau and α-synuclein) [5]. In particular, defective mitochondrial quality control (MQC) and loss of mitochondrial DNA (mtDNA) homeostasis have emerged as candidate pathogenic mechanisms triggered by oxidative damage in the setting of various age-related conditions [6,7,8]. Together with mitochondrial dysfunction, chronic inflammation seems to play a role in PD [4]. However, whether the two phenomena are inter-related is currently unclear.

Similar to other age-related conditions [9], the release of damage-associated molecules of different origins, including mitochondria, may provide a link between mitochondrial dysfunction and PD-associated systemic inflammation [10,11]. However, the mechanisms coordinating this multisystem process are unknown.

Extracellular vesicles (EVs) are delivery systems through which cells can communicate or remove unwanted materials. Among EVs, exosomes are intraluminal vesicles generated by the inward budding of small domains of early endosomal membranes producing intracellular multivesicular bodies (MVBs) [12,13,14,15]. MVBs undergo exocytic fusion and release their cargo into the extracellular space. Here, EV cargo may trigger inflammation [16]. The generation of mitochondrial-derived vesicles (MDVs) has been proposed as a further level of MQC operating via mitochondrial-lysosomal crosstalk [17].

Therefore, purification and characterisation of molecules packaged in and secreted through small EVs (sEVs)/exosomes into body fluids may provide meaningful biomarkers for the diagnosis and tracking of several disease conditions, especially those characterised by quality control disorders. Unveiling the pathways involving sEVs/exosomal trafficking also holds promise as a platform for developing novel therapeutic interventions. Indeed, the low immunogenic potential and the prion-like behaviour of exosomes, together with their ability to cross the blood–brain barrier, make these vesicles ideal nanodelivery systems for RNAi therapy [18], immunotherapy [19] and drug delivery carriers into the central nervous system [20,21].

Moving from these premises, we conceived the EXosomes in PArkiNson Disease (EXPAND) study with the aim to characterise the cargo of sEVs/exosomes isolated from the serum of PD patients to identify candidate biomarkers for PD.

## 2. Methods

### 2.1. Study Design and Population

The protocol of this case-control study was approved by the Ethics Committee of the Università Cattolica del Sacro Cuore (Rome, Italy) (protocol # 0045298/17). After obtaining written informed consent, a convenience sample of 40 participants, 20 cases (with a diagnosis of PD according to the Queen Square Brain Bank criteria [22]) and 20 controls (sex- and age-matched people without any signs of parkinsonism or potential premotor symptoms) was enrolled. The study was conducted in agreement with legal requirements and international norms (Declaration of Helsinki, 1964).

### 2.2. Participant Recruitment and Assessment

Participant recruitment took place at the Fondazione Policlinico Universitario “Agostino Gemelli” IRCCS (Rome, Italy) from March to November 2017 under the coordination of the Institute of Neurology at the Università Cattolica del Sacro Cuore. PD patients were men and women aged 70+ who had been under stable dopaminergic therapy for at least one month prior to enrolment [22]. Age- and gender-matched persons with no PD or family history of PD were enrolled as controls. Parkinsonism, progressive neurological diseases, potential premotor symptoms and dementia were considered exclusionary for both cases and controls.

Age, sex, smoking habit, functional status as assessed by the activities of daily living (ADL) [23] and instrumental ADL (IADL) [24] scales, comorbid conditions and medications were recorded for all study participants. Cognition was evaluated via the Mini Mental State Evaluation (MMSE) [25], whereas mood was assessed through the geriatric depression scale 15 items (GDS-15) [26].

Clinical characteristics of PD patients were assessed with the unified Parkinson’s disease rating scale (UPDRS) [27] and the Hoehn and Yahr staging [28] during the "on" state. The levodopa equivalent daily dose (LEDD, mg) was calculated according to published conversion factors for individual anti-parkinsonian drugs [29]. The LEDD is defined as the levodopa equivalent dose of a drug that produces the same symptomatic relief as 100 mg of immediate release levodopa (combined with a DOPA decarboxylase inhibitor).

### 2.3. Blood Sample Collection

Blood samples were collected in the morning by venipuncture of the median cubital vein after overnight fasting, using commercial collection tubes (BD Vacutainer^®^; Becton, Dickinson and Co., Franklin Lakes, NJ, USA). For serum separation, blood samples were left about 30 min at room temperature for clotting and subsequently centrifuged at 1000× *g* for 15 min at 4 °C. Aliquots of serum (0.5 mL/tube) were prepared from the upper clear fraction (serum) and then stored at −80 °C until analysis.

### 2.4. Exosomes Isolation and Characterisation

#### 2.4.1. Purification of Small Extracellular Vesicles/Exosomes by Differential Ultracentrifugation

**Figure 1:**
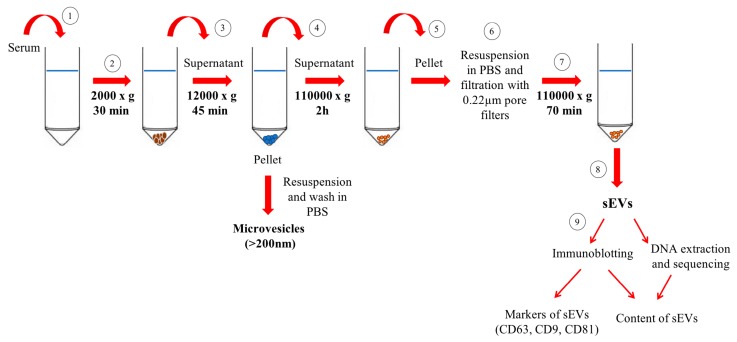
Schematic representation of the isolation and characterisation of small extracellular vesicles (sEVs) from serum. Serum is centrifuged at 2000× *g* at 4 °C for 30 min. Pellet is discarded to remove cell contamination (Steps 1 and 2) and supernatant is centrifuged at 12,000× *g* at 4 °C to remove apoptotic bodies, mitochondrial particles, cell debris and large vesicles (i.e., microvesicles with mean size >200 nm) (Step 3). Supernatant from Step 3 is ultracentrifuged for 2 h at 110,000× *g* at 4 °C (Step 4) and the pellet is collected (Step 5), resuspended in phosphate-buffered saline (PBS), filtered through a 0.22-μm filter (Step 6) and ultracentrifuged for 70 min at 110,000× *g* at 4 °C to eliminate contaminant proteins (Step 7). The pellet obtained from Step 7 is resuspended in 100 μL of PBS and represents purified sEVs (Step 8). After isolation, sEVs are analysed to confirm the presence of CD63, CD9 and CD81 markers and their content is characterised via immunoblotting and mitochondrial DNA sequencing analysis (Step 9).

Serum samples from PD patients and controls were diluted with equal volumes of phosphate-buffered saline (PBS) to reduce fluid viscosity. sEVs/exosomes were purified through differential centrifugation as described previously (Figure 1) [30,31,32]. Briefly, diluted samples were centrifuged at 2000× *g* at 4 °C for 30 min and pellets were discarded to remove any cell contamination. Subsequently, supernatants were centrifuged at 12,000× *g* at 4 °C for 45 min to remove apoptotic bodies, mitochondrial particles, cell debris and large vesicles (mean size >200 nm). Supernatants were collected and ultracentrifuged at 110,000× *g* at 4 °C for 2 h. Pellets were recovered and resuspended in PBS, filtered through a 0.22-μm filter and ultracentrifuged at 110,000× *g* at 4 °C for 70 min to eliminate contaminant proteins. Pellets enriched in purified sEVs/exosomes were resuspended in 100 μL of PBS. To quantify sEVs/exosomes, total protein concentration was measured using the Bradford assay [32]. The purity of sEV preparations was ascertained by transmission electron microscopy of randomly selected samples [32].

#### 2.4.2. Western Immunoblot Analysis of Small Extracellular Vesicles

Western immunoblot analysis will be carried out to determine the type of sEVs on the basis of expressed tetraspanins (CD63, CD9 and CD81) and to characterise their protein cargo [30]. Equal amounts of EV proteins from PD patients and controls will be separated by sodium dodecyl sulphate polyacrylamide gel electrophoresis (SDS-PAGE) and subsequently electroblotted onto polyvinylidenefluoride (PVDF) Immobilon-P (Millipore, Burlington, MA, USA). Afterwards, membranes will be probed with primary antibodies against CD9, CD63 and CD81 (Table 1). Mitochondrial markers will be assessed using a specific cocktail of antibodies (Table 1).

#### 2.4.3. Analysis of Mitochondrial DNA in Small Extracellular Vesicles

Purified sEVs/exosomes will be treated with 1 U of Baseline-ZERO DNase0 solution (Epicentre, Madison, Wisconsin) for 1 h at 37 °C to eliminate contaminating single and double-strand DNA adherent to the EV surface or present in solution. Enzyme inactivation will be obtained by incubation at 65 °C for 10 min [33]. After DNA extraction, mtDNA amplification will be achieved with the MitoALL resequencing kit (Applera, Norwalk, CT, USA) to identify mtDNA as fragments or whole genome in EVs [33,34].

Sanger sequencing of mtDNA will be carried out as previously described [35]. Prediction of pathogenic potential of missense mutations will be performed *in silico* by the freely available PolyPhen2 tool (Polymorphism Phenotyping v2; http://genetics.bwh.harvard.edu/pph2/), as previously described [36]. The automated pipeline MToolBox will be used to annotate mitochondrial variants and related features through the steps of read mapping, post-mapping processing, genome assembly, haplogroup prediction and variants annotation [37]. Finally, nucleotide site-specific variability will be evaluated using HmtDB [38] and HmtVar [39] databases.

### 2.5. Determination of Inflammatory Mediators

Markers of systemic inflammation will be assayed as previously described [18,40]. Briefly, a set of 27 pro- or anti-inflammatory mediators, including cytokines, chemokines and growth factors, will be measured in duplicate in serum samples using the Bio-Plex Pro™ Human Cytokine 27-plex Assay kit (#M500KCAF0Y, Bio-Rad, Hercules, CA, USA) on a Bio-Plex^®^ System with Luminex xMap Technology (Bio-Rad) (Table 2). Data will be acquired on a Bio-Plex Manager Software 6.1 (Bio-Rad) with instrument default settings and subsequently analysed. Outliers will be automatically removed by optimisation of standard curves across all analytes and results will be obtained as concentration (pg/mL).

### 2.6. Statistical Analysis

The strategy for the identification and validation of potential biomarkers for PD will rely on the building of discriminant models to differentiate cases from controls. The approach chosen for the EXPAND study will be based on partial least squares-discriminant analysis (PLS-DA) as previously described [41,42], because of its versatility and ability to deal with highly correlated predictors. The statistical reliability of the PLS-DA model will be verified by double cross-validation and through randomisation tests [43]. The identification of the experimental variables contributing the most to the classification model will be accomplished by inspection of variable importance in projection (VIP) indices [44] and rank product (RP) [45].

## 3. Discussion

Aberrant protein folding and accrual of protein aggregate, in particular α-synuclein, in midbrain dopaminergic neurons is a well-established pathogenic feature of PD.

Altered expression of genes encoding proteins involved in mitochondrial homeostasis [e.g., Parkin, PTEN-induced putative kinase 1 (PINK1), DJ-1, leucine-rich repeat kinase 2 (LRRK2), ATPase 13A2, vacuolar protein sorting-associated protein 35 (VPS35) is also linked to PD [46]. Indeed, mitochondrial dysfunction may contribute to protein misfolding via oxidative stress [5]. Impaired oxidative phosphorylation in PD seems to occur primarily as a consequence of complex I [47] and III deficiency [48], leading to increased production of reactive oxygen and nitrogen species.

In addition, oxidative stress and replication errors are responsible for the generation of mtDNA mutations in PD [49]. Partially deleted mtDNA molecules expand clonally because of replicative advantage over wild-type mtDNA and their accumulation results in complex IV deficit. Accrual of deleted molecules can be compensated for by increases in mtDNA content, but defective mtDNA homeostasis has been reported in PD [8,50].

Interestingly, non-neuronal changes accompany or even precede neurodegeneration during PD [2,3,4]. Indeed, systemic inflammation is associated with disease severity and rate of progression [4] but little is known about the molecular mechanisms linking inflammation with neuronal mitochondrial dysfunction.

The release of damage-associated molecules of different origins, including mitochondria, is an alleged mechanism connecting mitochondrial dysfunction and systemic inflammation during ageing as well as in the setting of various disease conditions [17]. In particular, the generation and release of MDVs have attracted considerable research interest.

**Figure 2:**
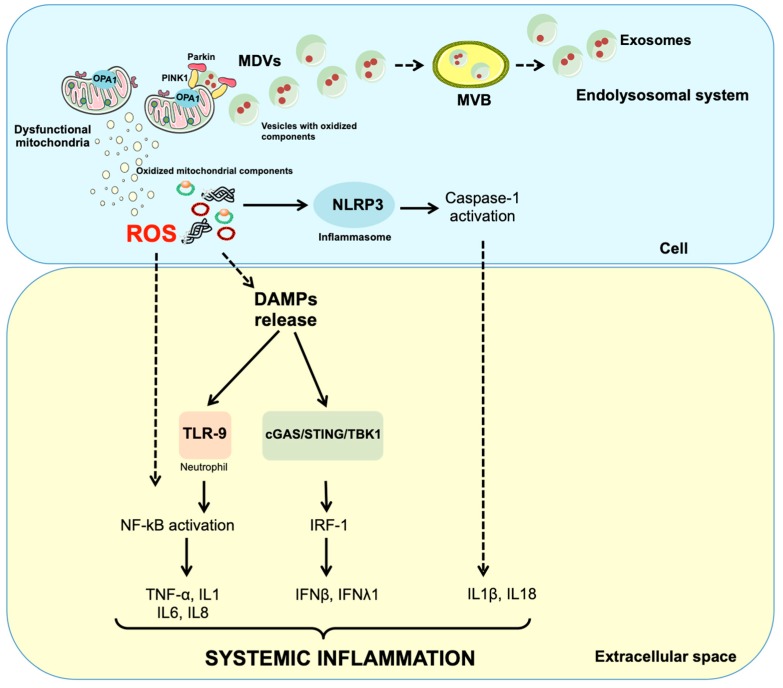
Schematic representation of possible mechanisms releasing mitochondrial components into the circulation. Dysfunctional but not yet depolarised mitochondria are targeted to degradation by serine/threonine-protein kinase PTEN-induced putative kinase 1 (PINK1) and Parkin. This priming process may also assist in the generation of mitochondrial-derived vesicles (MDVs). MDVs reach out the endolysosomal system and form multivesicular bodies (MVBs) that are unloaded outside the cell as exosomes. Impairment of mitochondrial quality control processes may also lead to accumulation of intracellular oxidised components that can be released as damage-associated molecular patterns (DAMPs) following a vesicle-free pathway. In particular, damaged mitochondrial transcription factor A (TFAM)-bound (green circles) or unbound (red circles) mitochondrial DNA (mtDNA) particles can be released as DAMPs. These molecules are pro-inflammatory and can activate three distinct signalling routes via interaction with (1) toll-like receptors (TLRs), (2) nucleotide-binding oligomerisation domain (NOD)-like receptor family pyrin domain containing 3 (NLRP3) inflammasome and (3) cytosolic cyclic GMP-AMP synthase (cGAS)-stimulator of interferon genes (STING) DNA-sensing system. IFN, interferon; IL, interleukin; IRF-1, interferon regulatory factor 1; NF-κB, nuclear factor κB; OPA1, optic atrophy 1; ROS, reactive oxygen species; TBK1, TANK-binding kinase 1; TNF-α, tumour necrosis factor alpha.

Albeit the mechanisms of MDV generation and cargo upload are still unclear, defective mitophagy might be crucial toward EV production in the central nervous system. Indeed, mildly damaged mitochondria are primed by serine/threonine-protein kinase PINK1 and Parkin and generate MDVs (Figure 2) [51]. Once formed, MDVs reach out of the endolysosomal system, forming MVBs, and are released into the extracellular compartment as exosomes [52]. As such, PINK1 and Parkin signalling provides a link between mitochondrial dysfunction and inflammation in PD.

Furthermore, under inflammatory conditions, recruitment of the cytosolic proteins Rab9 and Snx9 to mitochondria assists in mitochondrial antigen presentation and GTPase Rab7-induced fusion of MDVs with late endosomes for antigen processing [52]. This ensures the mounting and displaying of mitochondrial components by major histocompatibility complex I (MHC I) after proteolytic cleavage within the endoplasmic reticulum. These mitochondrial antigens at the cell surface may then activate specific cytotoxic T cells [53]. In dopaminergic neurons, the MHC I pathway is also elicited for endogenous peptide presentation [52,54]. Notably, MHC I presentation may also be induced by cytokine release from microglial cells activated by neuromelanin or α-synuclein. The latter instigates an immune response in mice [55,56]. By contrast, MHC II-deficient mice are protected against neuronal death under α-synuclein overexpression [57]. In PD patients, peptides derived from two different regions of α-synuclein (i.e., Y39, which is situated in close proximity to known α-synuclein point mutations, and S129) act as antigenic epitopes and stimulate an immune response by CD4+ or CD8+T cells [58].

Finally, mitochondrial stress provoked by partial depletion of mitochondrial transcription factor A (TFAM) can induce mtDNA escape from mitochondria to the cytosol where it engages innate immune signalling [59]. Circulating mtDNA contains hypomethylated CpG motifs typical of bacterial DNA that can trigger a sterile inflammatory response through the binding and activation of membrane or cytoplasmic pattern recognition receptors (PRRs), such as the Toll-like receptor (TLR), the nucleotide-binding oligomerisation domain (NOD)-like receptor (NLR) and the cytosolic cyclic GMP-AMP synthase (cGAS)-stimulator of interferon genes (STING) DNA sensing system-mediated pathways [60].

Taken as a whole, these studies suggest that mtDNA dyshomeostasis and MQC failure in PD may converge at the level of cell-mediated immunity.

An important limitation in the characterisation of EVs resides in the methods used for their isolation. Indeed, the term exosomes indicates either MVB-derived vesicles classified by their size (<150 nm) and the presence of CD63 or sEVs of undefined intracellular biogenesis, able to pass through 220-nm pore filters and recovered by high-speed centrifugation [61]. Current procedures for sEVs isolation yield a mixed population of sEVs, which impacts result interpretation.

Recently, Kowal and collaborators [62] examined different populations of sEVs purified after high speed ultracentrifugation and filtration through 220-nm pore filters and defined four subcategories of sEVs: i) sEVs positive for three tetraspanins, CD63, CD9 and CD81, enriched with endosomal markers and properly known as exosomes; ii) sEVs devoid of CD63 and CD81 but enriched with CD9 which are associated with plasma membrane and early endocytic signatures; iii) sEVs devoid of CD63, CD9 and CD81; and iv) sEVs characterised by the presence of extracellular matrix and serum-derived factors. These last two types of sEVs are devoid of endosomal markers and thus have a different, yet unknown origin. Therefore, before analysing their content and drawing conclusions, it is crucial to bear in mind such differences.

To overcome existing limitation, in EXPAND we have applied a protocol allowing isolation of type (i) and (ii) sEVs from serum to exclude vesicles of unknown origin. Isolated sEVs will be used for identification of mitochondrial components, in particular respiratory chain complex subunits and mtDNA and MQC factors (e.g., PINK1 and Parkin). These mediators have been chosen to provide a fairly comprehensive evaluation of EV cargoes possibly reflecting mitochondrial functional decay in PD, with a special focus on mtDNA analysis in reason of its pro-inflammatory properties [59].

Albeit proposing an innovative biomarker discovery methodology, our study is not devoid of limitations. The cross-sectional design will not allow for inferring about the time course of changes in biomarkers and the progression of PD over time or in response to specific interventions. Furthermore, the study is associative in nature and cause-effect relationship between the proposed biomarkers and PD pathophysiology cannot be inferred. Antiparkinsonian medications, in particular levodopa treatment, represent a confounding factor in metabolite profiling studies and care was taken in collecting serum at standardised time points.

## 4. Conclusions

The isolation and characterisation of sEVs/exosomes cargo have attracted considerable research interest for the identification of biomarkers to be used for the diagnosis and tracking of complex disease conditions. Furthermore, the low immunogenic potential and the prion-like behaviour of EVs together with their ability of crossing the blood–brain barrier make them ideal nanodelivery systems for RNAi therapy, immunotherapy and drugs delivery carriers into the central nervous system. The EXPAND study will provide the initial knowledge for the exploitation of EVs in PD management. The analysis of additional mediators may be pursued in future studies designed to gather mechanistic insights in PD pathophysiology. Indeed, according to the view of disease progression through spreading of noxious molecules, the analysis of misfolded proteins (e.g., α-synuclein) within vesicles may be highly relevant for diagnostic and treatment purposes.

## Figures and Tables

**Table 1:** Technical specifications of the primary antibodies for Western immunoblotting.

Antibody	Manufacturer and Catalogue Number	Type	Species	Detected Band MW (kDa)
ATP5A (complex V)	Abcam (ab1104413)	Monoclonal	Mouse	55
UQCRC2 (complex III)				48
MTCOI (complex IV)				40
SDHB (complex II)				30
NDUFB8 (complex I)				20
CD63	Santa Cruz Biotechnology (sc-5275)	Monoclonal	Mouse	26
CD81	Santa Cruz Biotechnology (sc-166020)	Monoclonal	Mouse	25
CD9	Santa Cruz Biotechnology (sc-13118)	Monoclonal	Mouse	25
MTCOII (complex IV)	Santa Cruz Biotechnology (sc-514489)	Monoclonal	Mouse	25
NDUFS3 (complex I)	Santa Cruz Biotechnology (sc-374283)	Monoclonal	Mouse	25
Parkin	R&D Systems (MAB14381)	Monoclonal	Mouse	52
PINK1	AbD Serotec (HCA150)	Monoclonal	Mouse	70
SDHA (complex II)	Santa Cruz Biotechnology (sc-390381)	Monoclonal	Mouse	70

ATP5A, adenosine triphosphate 5A; MTCOI, mitochondrial cytochrome C oxidase subunit I; MTCOII, mitochondrial cytochrome C oxidase subunit II; NDUFB8, NADH:ubiquinone oxidoreductase subunit B8; NDUFS3, NADH:ubiquinone oxidoreductase core subunit; PINK1, PTEN-induced putative kinase 1; SDHA, succinate dehydrogenase complex flavoprotein subunit A; SDHB, succinate dehydrogenase complex iron sulphur subunit B; UQCRC2, ubiquinol-cytochrome C reductase core protein 2.

**Table 2:** List of serum inflammatory biomarkers to be assayed by multiplex immunoassay.

Type	Biomarkers
**Cytokines**	IFNγ, IL1β, IL1Ra, IL2, IL4, IL5, IL6, IL7, IL8, IL9, IL10, IL12, IL13, IL15, IL17, TNF-α
**Chemokines**	CCL2, CCL3, CCL4, CCL5, CCL11, CXCL
**Growth factors**	FGF-β, GCSF, GMCSF, PDGF BB

CCL, C–C motif chemokine ligand; CXCL 10, C–X–C motif chemokine ligand 10; FGF, fibroblast growth factor; GCSF, granulocyte colony-stimulating factor; GMCSF, granulocyte macrophage colony-stimulating factor; IFN, interferon, IL, interleukin; IL1Ra, interleukin 1 receptor agonist; PDGF BB, platelet derived growth factor BB, TNF, tumour necrosis factor.

## Abbreviations

ATP5A	Adenosine triphosphate 5A
ADL	Activities of daily living
CCL	C-C motif chemokine ligand
cGAS	Cyclic GMP-AMP synthase
CXCL	C-X-C motif chemokine ligand
EV	Extracellular vesicle
EXPAND	EXosome PArkiNson Disease
FGF	Fibroblast growth factor
GCSF	Granulocyte colony-stimulating factor
GDS	Geriatric depression scale
GMCSF	Granulocyte macrophage colony-stimulating factor
IADL	Instrumental activities of daily living
IFN	Interferon
IL	Interleukin
IL1Ra	Interleukin 1 receptor agonist
LEDD	Levodopa equivalent daily dose
LRRK2	Leucine-rich repeat kinase 2
MDV	Mitochondrial-derived vesicle
MHC	Major histocompatibility complex
MMSE	Mini mental state evaluation
mtDNA	Mitochondrial DNA
MTCO	Mitochondrial cytochrome C oxidase subunit
MVBs	Multivesicular bodies
MQC	Mitochondrial quality control
NDUFB8	NADH:ubiquinone oxidoreductase subunit B8
NDUFS3	NADH:ubiquinone oxidoreductase core subunit
NLR	Nucleotide-binding oligomerisation domain-like receptor
NLRP3	NLR family pyrin domain containing 3
PBS	Phosphate-buffered saline
PD	Parkinson’s disease
PDGFBB	Platelet derived growth factor
PLS-DA	Partial least squares-discriminant analysis
PINK1	Serine/threonine-protein kinase PTEN-induced putative kinase 1
PRRs	Pattern recognition receptors
PVDF	Polyvinylidenefluoride
RAB	Ras-related protein in Brain
RNS	Reactive nitrogen species
ROS	Reactive oxygen species
RP	Rank product
SDH	Succinate dehydrogenase complex flavoprotein subunit
SDS-PAGE	Sodium dodecyl sulfate polyacrylamide gel electrophoresis
sEV	Small extracellular vesicle
STING	cGAS-stimulator of interferon genes
TFAM	Mitochondrial transcription factor A
TLR	Toll-like receptor
TNF	Tumor necrosis factor
UPDRS	Unified Parkinson’s disease rating scale
UQCRC2	Ubiquinol-cytochrome C reductase core protein 2
VIP	Variable importance in projection
VPS35	Vacuolar protein sorting-associated protein 35
